# Does Fire Influence the Landscape-Scale Distribution of an Invasive Mesopredator?

**DOI:** 10.1371/journal.pone.0107862

**Published:** 2014-10-07

**Authors:** Catherine J. Payne, Euan G. Ritchie, Luke T. Kelly, Dale G. Nimmo

**Affiliations:** 1 Centre for Integrative Ecology, School of Life and Environmental Sciences, Deakin University, Melbourne, Victoria, Australia; 2 Australian Research Council Centre of Excellence for Environmental Decisions, School of Botany, University of Melbourne, Melbourne, Victoria, Australia; University of Regina, Canada

## Abstract

Predation and fire shape the structure and function of ecosystems globally. However, studies exploring interactions between these two processes are rare, especially at large spatial scales. This knowledge gap is significant not only for ecological theory, but also in an applied context, because it limits the ability of landscape managers to predict the outcomes of manipulating fire and predators. We examined the influence of fire on the occurrence of an introduced and widespread mesopredator, the red fox (*Vulpes vulpes*), in semi-arid Australia. We used two extensive and complimentary datasets collected at two spatial scales. At the landscape-scale, we surveyed red foxes using sand-plots within 28 study landscapes – which incorporated variation in the diversity and proportional extent of fire-age classes – located across a 104 000 km^2^ study area. At the site-scale, we surveyed red foxes using camera traps at 108 sites stratified along a century-long post-fire chronosequence (0–105 years) within a 6630 km^2^ study area. Red foxes were widespread both at the landscape and site-scale. Fire did not influence fox distribution at either spatial scale, nor did other environmental variables that we measured. Our results show that red foxes exploit a broad range of environmental conditions within semi-arid Australia. The presence of red foxes throughout much of the landscape is likely to have significant implications for native fauna, particularly in recently burnt habitats where reduced cover may increase prey species’ predation risk.

## Introduction

Predators shape ecosystems worldwide [1]. They can exert top-down regulation of lower trophic levels [2] and induce trophic cascades which flow through entire ecosystems [3]. Predators introduced to areas outside of their native range can have a particularly strong effect on native species [4], and have caused population declines and extinctions in a range of ecosystems [5]. Many invasive predators are ‘mesopredators’: smaller predator species that increase in abundance or activity following the removal of apex predators [6]. For example, in Australia, persecution of the native apex predator, the dingo (*Canis dingo*), has led to increases in the density or activity of invasive mesopredators (e.g. the red fox [*Vulpes vulpes*]) throughout large portions of the continent [3].

Fire is another globally significant process that affects environments worldwide [7]. Fire influences ecosystems via bottom-up control by altering the availability of key resources for biota. Fire incinerates plant matter, altering vegetation structure [8], [9], which in turn affects the distribution and abundance of animals [10].

Invasive mesopredators and fire share an important characteristic from a conservation perspective: both can be manipulated through management interventions. Invasive mesopredators are managed using lethal control and exclusion fencing, and fire using suppression or prescribed burning. However, management of mesopredators and fire usually occurs in isolation, without consideration of the potential effects of fire *on* mesopredators [11]. It is important to rapidly address this significant knowledge gap because some fire regimes may exacerbate the effects of invasive mesopredators by simplifying vegetation and amplifying predation risk [12], [13]. For example, interactions between fire regimes and invasive mesopredators have been hypothesised as a cause of lower survival of reptile species in recently-burned areas [14], and a contributor to the collapse of small mammal communities in northern Australia [15].

The red fox is one of the world’s most widely distributed mesopredators. It is common in both the northern and southern hemispheres. Foxes, and a second introduced mesopredator, the feral cat (*Felis catus*), are widely regarded as the primary cause of extinctions and declines of Australia’s marsupial fauna [5]. Evidence for the negative impact of foxes has been demonstrated through predator-control experiments that have shown that prey species increase in both range and activity when foxes are removed [16], [17]. Further evidence comes from dietary studies showing foxes eat a wide range of native mammal, reptile, bird, and invertebrate prey [18]–[20].

Despite indications that foxes may inhibit the recovery of native species following fire [12], [21], whether foxes are themselves influenced by fire remains poorly known. This knowledge gap limits the ability of land managers to consider the effects of fire management on red foxes, which could have negative ramifications for native biodiversity. While foxes are widely considered as habitat generalists, they do display local variability in occurrence related to habitat or landscape structure [22]. For example, in some regions, foxes prefer heterogeneous landscapes [22], as they are able to use multiple landscape elements on a daily or seasonal basis [23], [24]. Fire management in many regions seeks to maximise landscape heterogeneity by creating mosaics of fire ages (i.e. ‘patch mosaic burning’; [25]). Does such management inadvertently favour invasive mesopredators?

The few studies that have explored the topic have focused on relatively short temporal scales (<30 years and often <10 years post fire) or small spatial scales (but see [26]). However, in some ecosystems, post-fire vegetation recovery continues for a century or more after fire [27]. Consequently, animal species respond to fire over similarly long time-frames [28]. The effects of fire can also occur across multiple spatial scales [29]; while time since fire may affect a species’ occurrence at any *point* in the landscape, the area and composition of fire-ages within a ‘whole’ landscape can play a critical role in affecting species’ landscape-level distributions [30]. This is likely to be especially true for large, mobile species, such as the red fox.

In addition to the effects that fire may have on species’ occurrence, other environmental factors may be locally important. With regard to foxes, this includes climate [26], the distribution of vegetation types [31], and the distance to roads [24] and agricultural land [22]. Foxes rely on free standing water for drinking, particularly when temperatures are high (>30°C), as is common in many semi-arid environments. Hence, as annual rainfall decreases (aridity intensifies) permanent water may be reduced in its availability and limit fox occurrence. Foxes are often thought of as edge specialists [22]. They often prefer to hunt in open areas such as resource-rich agricultural fields or structurally simple vegetation types adjacent to more complex vegetation which provides cover during the day [22], [32]. Their ability to hunt may be further enhanced where roads create easy access and increased visibility in otherwise structurally complex habitats [24], [33].

Here, we examine what drives the occurrence (reporting rate) of red foxes in semi-arid Australia at multiple spatial scales, with a particular emphasis on the role of fire. We conducted two large-scale natural experiments. First, we explored landscape-scale patterns of fox occurrence in relation to the properties of fire mosaics; namely, the amount and diversity of fire age-classes within each of 28 study landscapes (each 12.6 km^2^). Second, we explored site-scale patterns of fox occurrence in relation to fire history at 108 sites stratified along a century-long post-fire chronosequence. In both cases, we also quantified the influence of other environmental variables such as vegetation type and distance to agricultural land. Our aims were: 1) to determine the drivers of fox distribution in semi-arid Australia; and 2) to understand the specific role of fire in influencing fox occurrence at large scales relevant to fire and mesopredator management.

## Materials and Methods

### Study region

**Figure 1:**
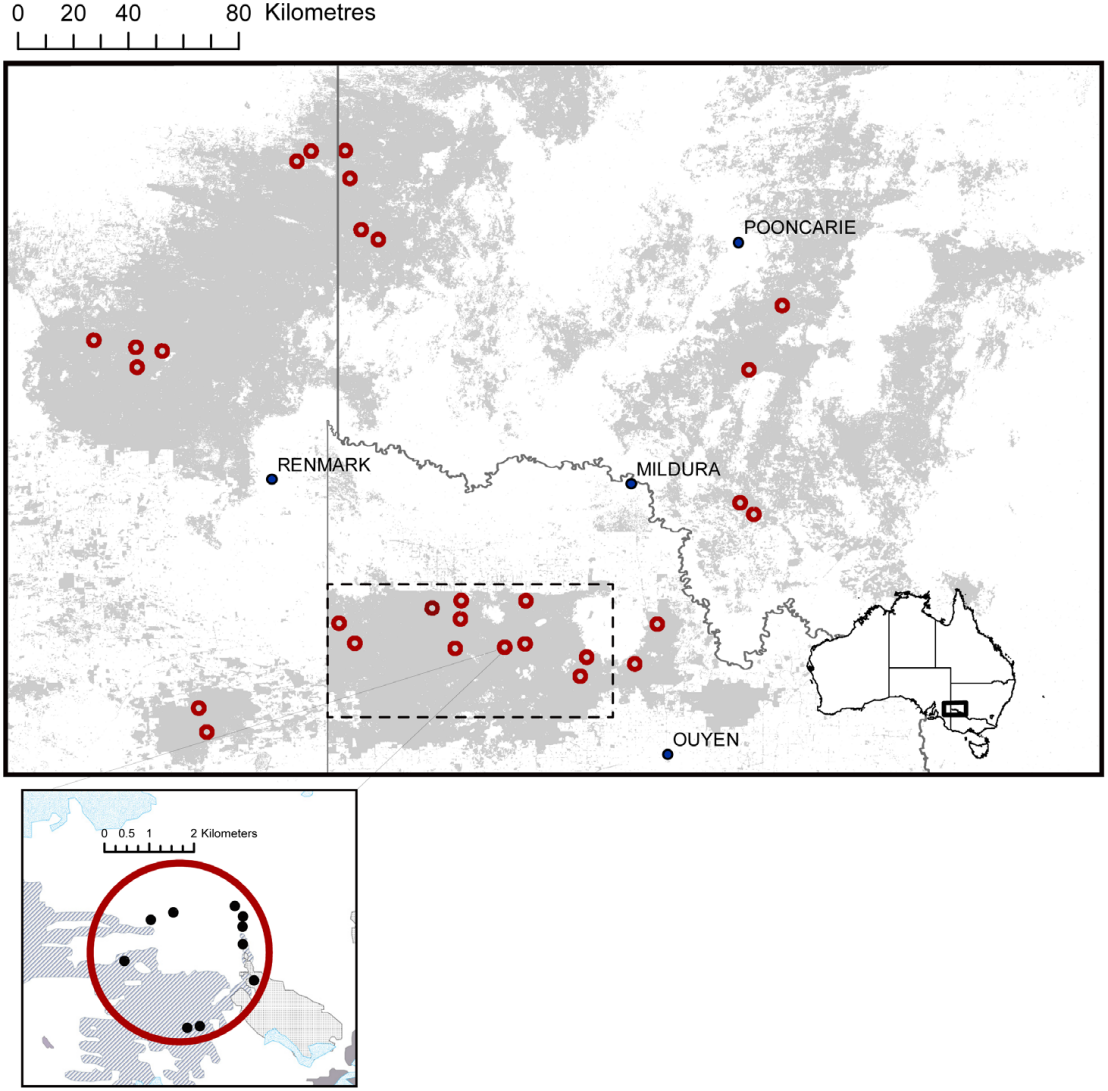
Map of study area showing all landscapes (circles) considered in this study (grey shading indicates mallee vegetation; majority of white areas indicates agricultural land used for grazing and cereal crops). The dashed box shows the spatial extent of the site-scale study. An inset shows an example of a study landscape including the position of 10 sites within where site-scale data were collected. Within the inset, different hatching represents different fire ages.

This study was undertaken in the Murray Mallee region of south-eastern Australia (Fig. 1). The climate in the region is semi-arid, with mean annual rainfall of 200–350 mm and average daily maximum temperatures are 30–33°C in summer and 15–18°C in winter (Australian Bureau of Meteorology; http://www.bom.gov.au). The vegetation is predominantly ‘tree mallee’ characterised by an overstorey of *Eucalyptus* species (<5–8 m) with a multi-stemmed growth form [34]. Two vegetation types are common throughout region [35]. ‘Triodia Mallee’ has a canopy of *Eucalyptus dumosa* and *E. socialis* with an understorey of *Triodia scariosa* and mixed shrubs, and occurs mainly on sandier soils typical of dunes. ‘Chenopod Mallee’ has a canopy of *E. oleosa* and *E. gracilis* with an open understorey of chenopod species, and occurs on heavier soils typical of swales.

**Figure 2:**
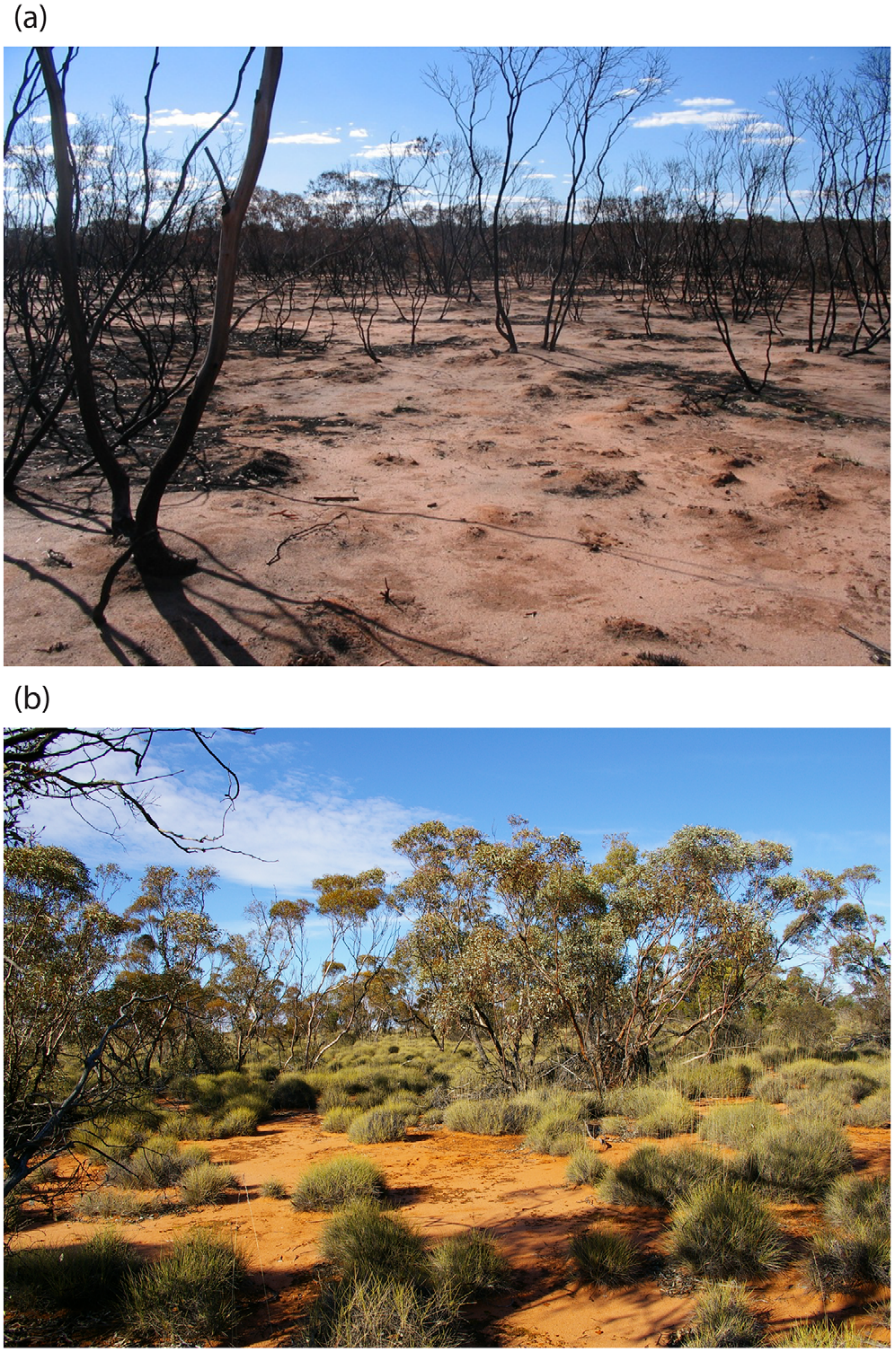
Examples of mallee vegetation with differing fire histories. (a) A recently burned site; (b) A long unburned site.

Mallee vegetation is fire-prone with large fires (i.e. >100,000 ha) occurring somewhere in the region on a bidecadal basis [36], although individual sites can go long periods without fire (i.e. >100 years; [37]). Fire is actively managed in the region through prescribed burning and suppression for both asset protection and conservation objectives [36]. Most wildfires are ignited by lightning strikes and are stand-replacing, essentially resetting vegetation succession to ‘year-zero’ (Fig. 2; [8]).

### Site selection

We refer to two datasets in this study derived from two different natural experiments that differed in both their spatial grain and extent. We refer to these as ‘landscape-scale’ and ‘site-scale’ datasets throughout, in reference to the spatial scale of the response and predictor variables (i.e. the spatial grain) of the respective datasets.

#### Landscape-scale data

The landscape-scale dataset consists of 28 study landscapes, each with a 4 km diameter circle (12.6 km^2^; Fig. 1), distributed throughout a 104, 000 km^2^ study area. These landscapes were selected as part of a broad-scale natural experiment: the Mallee Fire and Biodiversity Project. Study landscapes were selected to allow a comparison of the effects of different approaches to patch mosaic burning on biodiversity, with a particular emphasis on the role of the area and diversity of fire-ages (‘pyrodiversity’, see [25]). Thus, landscapes were stratified according to number and spatial extent of fire-age classes within the landscape [29]. The fire history of the region was mapped using the ENVI package [38] and then converted to shape files for use in ArcMap version 9.2 [39]. Only fires that occurred post-1971 were mapped due to limited availability of Landsat imagery prior to this time (see [36]).

#### Site-scale data

We collected site-scale data within a subset of the 28 study landscapes located within the region’s largest national park; Murray Sunset National Park (6630 km^2^; Fig. 1). Ten sites were established within each of 10 of the original study landscapes. Sites were distributed to incorporate a range of fire-age classes (range = 7–105 years), as well as capturing geographic and topographic variation. We established an additional landscape, containing 12 sites, following an experimental burn during the study (fire age = 0 years), resulting in 11 landscapes containing 112 sites. We omitted four sites to comply with ethics permits due to their close proximity to active nesting sites of the endangered malleefowl (*Leipoa ocellata*). This resulted in a total of 108 sites being surveyed. All sites were a minimum of 200 m apart and typically >100 m from the edge of a fire-age class.

### Predator surveys

#### Landscape-scale data

We surveyed large mammalian predators using track surveys from three sand-plots within each study landscape (n = 84 in total). Each sand-plot was a 100 m×2 m area smoothed out by dragging a weight along an unsealed vehicle track. The locations of sand-plots within landscapes were chosen to incorporate variation in the topography (dunes and swales) within each landscape. Sand-plots were typically >500 m apart. We checked each sand-plot for tracks once per day by walking along the transect and identifying tracks to species level for three consecutive days in spring (October–November 2007), and again in summer (January–March 2008), resulting in six survey nights for each sand-plot and thus 18 survey nights per landscape. Following checking, sand-plots were smoothed over in preparation for the following day. If the sand-plot was heavily disrupted on one day (due to weather or vehicle disturbance), it was surveyed for an additional day.

#### Site-scale data

We used camera traps (Passive ScoutGuard 550; ScoutGuard IR Cameras, Australia) to survey for mammalian predators at the site-scale during April–July 2012. We installed one camera per site and deployed each for a minimum of 15 nights. We attached cameras to a post at a height of 0.5 m and positioned them facing southward. Vegetation was removed within the immediate area of the camera to reduce false triggering. A 15 second video was taken each time the camera motion sensor was triggered. To attract predators to the front of the camera from the local vicinity, we placed a scent lure of tuna oil soaked into chemical wadding inside a bait holder made from PVC piping with steel mesh at one end. We positioned the lure 3 m from the base of the camera post, and secured it to the ground with a peg.

### Predictor variables

#### Landscape-scale data

Six predictor variables were chosen to represent the properties of the study landscapes (Table 1). Three of these variables represent the fire history of the landscape: (1) the extent of recently burnt vegetation in the landscape (<10 years since fire; ‘recently burned’); (2) the extent of long unburnt vegetation (unburned since 1972; ‘long unburned’); and (3) the diversity of fire-ages within a landscape (‘fire diversity’). Fire diversity was calculated as the Shannon-Wiener diversity index of the proportional cover of fire age-classes within each landscape.

**Table 1:** Predictor variables included in models using the landscape-scale and the site-scale datasets.

Dataset	Predictor variable	Description
***Landscape-scale data***	Recently burned	Extent of landscape burned within 10 years of surveys
	Long unburned	Extent of landscape not burned since 1972 (>35 years since fire)
	Fire diversity	Shannon-Wiener diversity index of the extent of three fire age classes (0–10 years, 11–35 years and >35 years)
	Solar radiation	Long-term average monthly gridded solar exposure (MJ/m^2^) from 1990–2008 for each landscape
	Triodia Mallee	Extent of landscape comprised of vegetation type in which *Triodia scariosa* typically occurs
	Distance to agricultural land	Distance from the centre of each landscape to contiguous non-mallee vegetation (m)
***Site-scale data***	*Time since fire*	Amount of time since a site last experienced fire (years)
	Bare ground cover	Cover of bare ground present
	*Triodia* cover	Cover of *Triodia scariosa* <1 m
	Eucalypt cover	Cover of eucalypt shrubs <1 m
	Shrub cover	Cover of non-eucalypt shrubs <1 m
	Vegetation type	Broad vegetation classification (Triodia Mallee or Chenopod Mallee)
	Distance to edge	Distance from each site to the nearest park boundary (m)
	Distance to road	Distance from each site to the nearest road (m)

Three predictor variables were chosen to describe properties of the study landscapes other than fire history. We used a measure of mean solar radiation (‘solar radiation’) as a surrogate for aridity across the region. The solar radiation variable represents the total amount of solar energy falling on a horizontal space per day (MJ/m^2^). We derived these values from a gridded data set (5 km resolution) extending over 18 years (1990–2008; Australian Bureau of Meteorology http://www.bom.gov.au, 2009). Solar radiation was the mean of the 18 yearly averages of the grids that overlaid each landscape. Solar radiation is negatively correlated with annual rainfall and positively correlated with temperature. We used the proportional extent of *Triodia* mallee vegetation (‘Triodia Mallee’) within the landscape to capture differences in vegetation types. The extent of mallee vegetation in the study area was mapped in previous work (see [35]). Finally, we used the distance from the centre point of each landscape to the closest area of contiguous non-mallee vegetation (‘distance to agricultural land’), to capture the context of landscapes with respect to landscape modification. The area surrounding each reserve is comprised almost entirely of grazing land and grain crops. We calculated distance to agricultural land using ArcGIS [39].

#### Site-scale data

Eight predictor variables were chosen at the site level (Table 1). The fire history of sites was represented by the time since the last fire (‘time since fire’; range: 0–105 years). This was determined using two methods. Recent fire history (since 1972) was calculated using the fire history maps (see [36]). Fire-ages for sites burnt prior to the availability of satellite imagery (i.e. before 1972) were estimated using regression models of the relationship between stem diameter and tree age, and then using stem diameter to estimate the age of trees in areas where fire history was unknown (see [37] for detailed methods). This extended the time since fire axis from 0–32 years to 0–105 years.

Vegetation type was considered as a categorical variable with two levels: Triodia Mallee or Chenopod Mallee (‘vegetation type’). We again considered the effects of landscape modification by including the distance of sites to both the border of the National Park (1.72–21.28 km; ‘distance to edge’) and dirt roads (range: 28–1044 m; ‘distance to road’). We used park boundary as a proxy for an edge habitat because the park forms abrupt boundaries with cleared agricultural land and other non-mallee vegetation. We calculated distance variables using ArcGIS [39]. Aridity (solar radiation) was not considered at this scale as the data were collected from a single reserve.

Four additional predictor variables were included to describe vegetation structure at the sites. We established vegetation transects in representative areas 15 m from each camera location. We recorded substrate type and vegetation structure at 1 m intervals along a 50 m transect using a 2 m structure pole (2 cm diameter) held vertically above the ground. The four variables considered in the analysis represent the cover of open, bare ground (‘bare ground cover’), spinifex (‘*Triodia* cover’), eucalypt shrubs (defined as Eucalypt trees <3 m in height ‘eucalypt cover’) and non-eucalypt shrubs (‘shrub cover’). Bare ground was included because it gives an approximation of the ‘openness’ of the vegetation at the ground level. The cover of spinifex, eucalypt shrubs, and non-eucalypt shrubs were included as they form the majority of the ground and understorey structural complexity, and are known to drive fauna in the region [34], [40].

### Response variables

For both datasets, the response variable was the ‘reporting rate’ of foxes. At the landscape-scale, we defined reporting rate as the number of nights that a fox was recorded as ‘present’ and ‘absent’, respectively, at a sand pad over the 18 nights of sampling per landscape (i.e. three sand-plots surveyed for six nights in each landscape). Likewise, at the site-scale, reporting rate is the number of nights that foxes were and were not detected at the site, respectively, over the course of sampling (i.e. 15 nights).

### Statistical analysis

We used generalised linear mixed models (GLMMs) with the Laplace approximation [41] to examine the relationship between response and predictor variables at both landscape and site-scales. In landscape-scale models, we included ‘reserve’ as a random effect to account for spatial clustering of landscapes in conservation reserves (Fig. 1). Similarly, in the site-scale models, we included ‘landscape’ as a random effect to account for potential spatial correlation due to the clustering of sites into landscapes. Because we were studying the reporting rate of red foxes, a proportion, we modelled the response variable (at both scales) using a binomial distribution of errors and a logit link function.

For the landscape-scale dataset, we developed a set of candidate models that included all combinations of the six landscape-scale predictor variables. At the site-level, we developed two separate sets of models. As fire affects the variables used to describe vegetation structure (e.g. *Triodia* cover, bare ground cover; [8]), including both fire and vegetation structure variables in the same model could result in unreliable parameter estimates due to colinearity between predictor variables [42]. Thus, one model set (model set 1) included time since fire, vegetation type, distance to edge and distance to road, and a second model set (model set 2) included the vegetation structure variables (bare ground cover, *Triodia* cover, eucalypt cover and shrub cover). All combinations of predictors within the two sets of models were considered, meaning all eight site-level variables were in the same number of models overall. All variables included within a model set had low levels of colinearity (i.e. *r* <0.5). We tested both datasets for overdisperson using Pearson’s residuals [43], and found no evidence of overdispersion.

We compared each set of candidate models using Akaike’s Information Criterion corrected for small sample sizes (AICc; [44]). To compare the level of support for each model relative to the most parsimonious model, we calculated the difference (Δ*_i_*) between the AIC_c_ value of the best model (lowest AIC_c_ value) and the AIC_c_ value of each candidate model [44]. We considered models with Δ*_i_*<2 to have substantial support [44]. We also calculated the Akaike weight (*w_i_*) for each model. By summing these weights to calculate predictor weights (∑*w_i_*) for each variable, we were able to explore the influence of individual predictor variables at both the landscape and site level.

When there was no clear ‘best model’ (i.e. the most parsimonious model was not strongly weighted [*w_i_*<0.9]), we used model averaging to determine the direction and magnitude of the effect of each predictor variable [44]. We considered a variable as important when the associated 95% confidence interval of the averaged estimate did not overlap with zero. We performed all statistical analyses in R version 2.15.1 [45] using the lme4 package [41] and the MuMIn package [46].

### Ethics statement

The landscape-scale data were collected with approval from animal ethics committees at La Trobe University (approval number AEC06/07[L]V2) and Deakin University (approval number A41/2006), and permits from the Department of Sustainability and Environment, Victoria (permit 10003791), the Department of Environment and Heritage, South Australia (permit 13/2006), and the National Parks and Wildlife Service, NSW (license number S12030). The site-scale data were collected in accordance with the regulations of the Deakin University Animal Ethics Committee (approval number B10-2012) and in accordance with Department of Sustainability and Environment, Victoria (approval number 10006279).

## Results

At the landscape-scale, we recorded fox tracks in 24 of 28 (86%) study landscapes. We detected foxes on 3.32±0.49 (mean ± standard error) of 18 nights per landscape over the total sampling period. Other large-bodied, mammalian predators were uncommon: we detected cats at only 7 of 28 landscapes (25%). At the site-scale, we observed foxes at 62 of 102 (61%) sites (six cameras failed to reach the full 15 day survey period due to fault and were excluded from further analysis i.e. n = 102) and found the species to be widely distributed across the study area. We did not detect any cats at the site-scale over the 15 night sampling period.

At the landscape-scale, all models were a poor fit for the data and explained <6.5% of the variation in the data (% deviance explained). At the site-scale, all models explained <3.5% of the variation in the data. For both datasets, model selection indicated there was a similar level of support for several models (Δ*_i_*<2; Table 2), including the intercept-only model (i.e. only an intercept terms, no predictor variables), which received substantial support at both scales. As no single model was supported as being clearly best (i.e. *w_i_*>0.9; Table 2), we employed multi-model inference using model averaging to estimate the size, direction and uncertainty of parameter effects for fox explaining reporting rate in both datasets.

**Table 2:** Model selection results for red fox reporting rate for landscape-scale and sits-scale datasets.

Candidate model	df	LogLik	AIC_c_	Δ*_i_*	*w_i_*	%Dev
*Landscape-scale dataset*						
Null model (intercept only)	2	−32.37	69.2	0.00	0.14	0.00
Distance to agricultural land	3	−31.21	69.4	0.21	0.12	3.57
Distance to agricultural land + Triodia Mallee	4	−30.53	70.8	1.58	0.06	5.68
Triodia Mallee	3	−31.99	71.0	1.76	0.06	1.17
Fire diversity	3	−32.10	71.2	1.99	0.05	0.82
*Site-scale dataset*						
Bare ground cover	3	−65.90	138.0	0.00	0.13	2.16
Bare ground cover + *Triodia* cover	4	−65.07	138.6	0.51	0.10	3.39
*Triodia* cover	3	−66.26	138.8	0.73	0.09	1.62
Null model (intercept only)	2	−67.36	138.8	0.79	0.09	0.00
Bare ground cover + eucalypt cover	4	−65.67	139.8	1.71	0.06	2.50

Models are shown for which Δ*_i_*<2.0.

**Figure 3:**
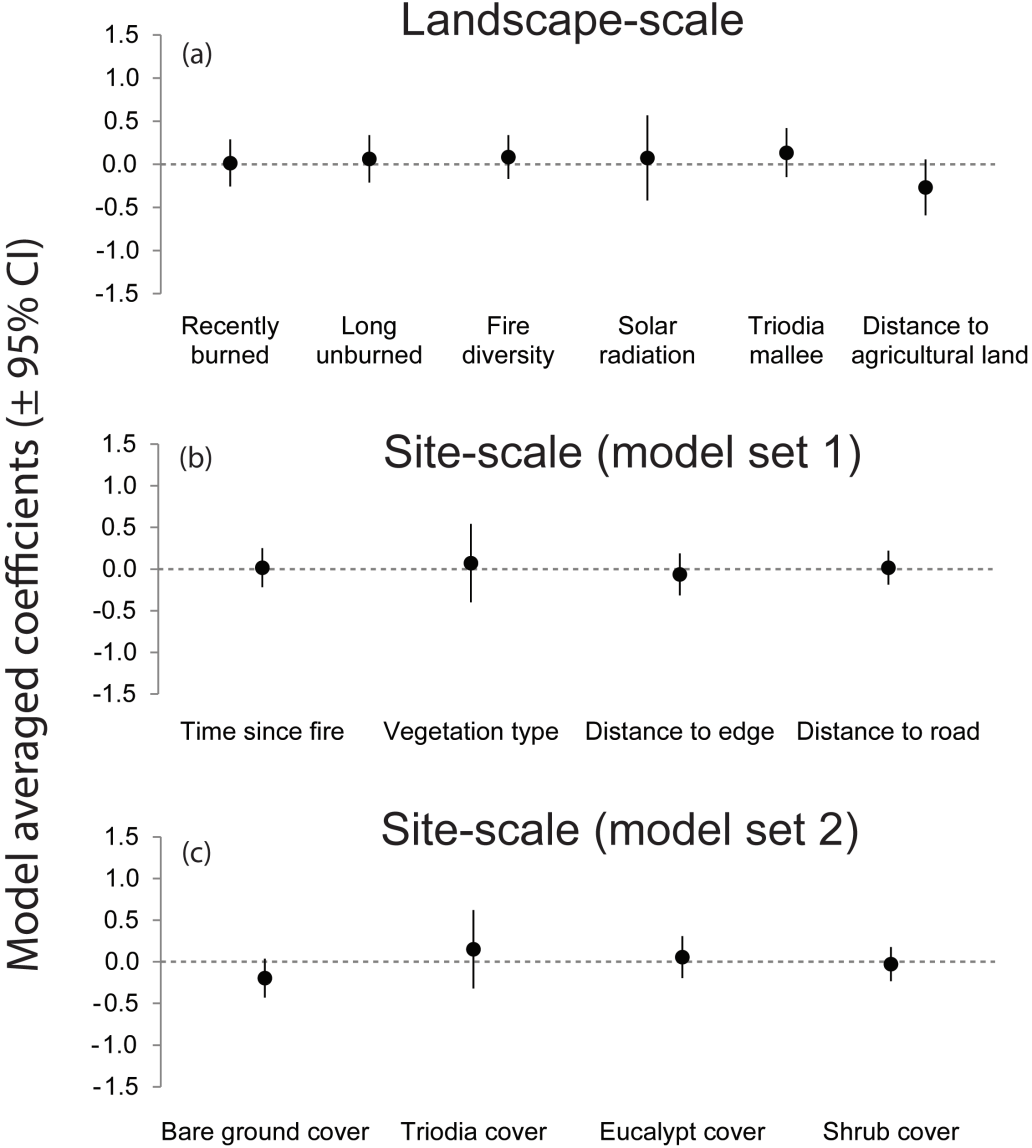
Model-averaged regression coefficients and 95% confidence intervals of models describing the reporting rate of foxes at both the landscape-scale (a) and site-scale (b and c).

The model-averaged coefficients for each predictor variable, in both datasets, were small and uncertain. The 95% confidence intervals of all predictor variables overlapped with zero (Fig. 3). The ∑*w_i_* for all predictor variables was low: <0.5 and <0.6 for the landscape- and site-scale datasets respectively.

**Figure 4:**
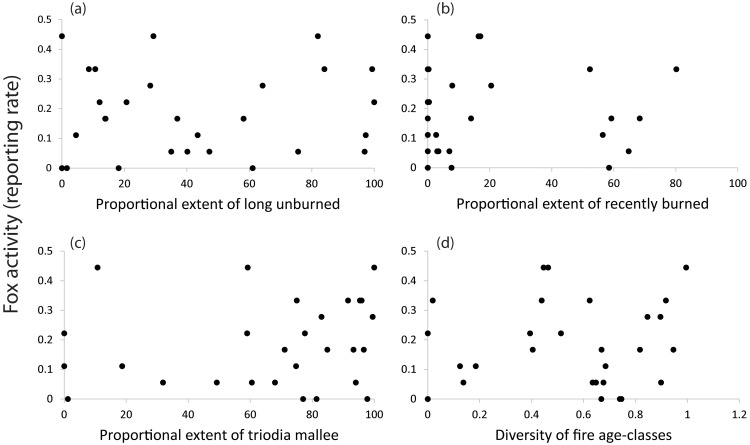
Relationships between the reporting rate of the red fox and the properties of fire mosaics. Circles are raw data points.

Graphical exploration of the data further highlights that fox activity was not strongly linked to key predictor variables (Fig. 4). In summary, the data shows that neither fire, nor any other predictor variable measured, affected the reporting rate of foxes at either the landscape- or site-scale.

## Discussion

Introduced mesopredators and fire are two processes that shape ecosystems around the world [4], [7]. Here, we have shown that a widespread and ecologically devastating mesopredator, the red fox [5], is largely unaffected by fire and is an extreme habitat generalist in semi-arid Australia. This result was confirmed using two large, complementary datasets, collected at different times and characterised by differing spatial scales and sampling strategies.

### Fire and the red fox

Our findings show that fire does not exert a strong influence on the distribution of the red fox in semi-arid mallee ecosystems. Despite conducting two intensive natural experiments across a broad geographic region, we did not detect a relationship between the reporting rate of foxes and fire history at either the landscape- or site-scale. At the landscape-scale, the red fox was recorded equally often in landscapes dominated by recently burned or long unburned vegetation, and in landscapes with a single fire age-class as those with a diversity of fire ages. At the site-scale, the red fox has a similar reporting rate in recently burned sites as in sites unburned for over a century. The post-fire preferences of the red fox are thus extremely broad, both spatially and temporally (also see [12], [47]).

Fire causes significant changes to vegetation structure over century-long time frames in mallee ecosystems [8]. In doing so, fire affects the distribution of a large range of fauna species [34]. Indeed, work conducted within the same study landscapes has shown the large and long-term effects fire has on birds, reptiles, and small mammals [28]–[30]. The lack of a response to fire by foxes is therefore not typical of native fauna in the region. It also suggests that foxes are not restricted to areas with particular soil or vegetation attributes for denning. This is consistent with foxes not being affected by any of the vegetation attributes measured (e.g. *Triodia* cover, shrub cover etc.).

A related way that fire could influence foxes is by altering the distribution of prey resources. As mentioned above, the distribution of many prey species are significantly affected by fire in the study region (e.g. birds, mammals, reptiles). Thus, foxes occupy a range of post-fire ages despite the strong influence of fire on the type and abundance of prey available. Red foxes have a broad and generalist diet [48], being able to consume a wide range of prey including both vertebrates and invertebrates, and even vegetation [19], [20]. Furthermore, foxes are capable of prey switching to capitalize on the most abundant prey source available [18], [49], thereby reducing their reliance on any particular prey item. This flexibility in their diet is likely to be a key component of their life history that allows them to occur within such a broad range of post-fire conditions.

One objection to our findings at the site-scale may be that the local site is not a relevant spatial scale to characterize the effects of fire, as foxes are a relatively large and mobile species. Given the large estimated home ranges of foxes in other parts of arid Australia (e.g. 8–33 km^2^; [31]), foxes may select broader areas (i.e. kms^2^) that capture their resource requirements across entire landscapes, and this might include a large area of a particular fire-age, or multiple fire ages. Such use of multiple habitat types by foxes has been demonstrated in other systems [23], [24]. Our landscape-scale study characterized land mosaics at a large scale relevant to the home range of foxes (12.6 km^2^), and still failed to detect any relationship between fox activity and fire history. Therefore, our results suggest that the lack of relationships between fox reporting rate and fire history does not stem from spatial scaling issues. Instead, foxes are resilient towards the effects of fire at multiple temporal and spatial scales.

### Climate and distance to modified land

In addition to fire, we examined other variables that could influence the distribution of the red fox. Here, we again found red foxes to be flexible to a broad range of ecological conditions. Foxes displayed no response to an aridity gradient across the study region. This lack of response to aridity is unsurprising, as the geographic range of the red fox spans the northern hemisphere and much of Australia, suggesting the species is capable of coping with a range of climatic conditions.

Despite foxes occupying a broad climatic niche in space, fluctuations in populations do occur in response to extreme weather events. For example, fox populations in arid areas rise rapidly following high rainfall events, in response to increased prey availability [50]. Our site-scale study was carried out during a year of record high rainfall (Australian Bureau of Meteorology, Ouyen Station). Considered in isolation, this may suggest that the wide distribution of the fox was partly due to a productivity-related increase in food resources (predominantly populations of native and introduced rodents; [40]). However, the landscape-scale data were collected near the end of a severe, decade-long drought. Foxes were widely distributed across the region despite the drought. This indicates that, in semi-arid Australia, foxes can be widespread during a broad range of climatic conditions and despite fluctuations in their prey populations which accompany climactic extremes [40].

Some studies have found that foxes are positively associated with edges between fragments and modified land (e.g. agricultural land) [22], [32]. Our results indicate that foxes do not show a preference for edge habitats in mallee ecosystems, despite our sites and landscapes capturing a broad gradient of distances to agricultural land, from <2 km to >30 km. Edge habitats may be more important for foxes in highly fragmented landscapes, where they occur with small remnant patches of wooded vegetation which provide the only available cover [22]. While the mallee region has been subject to large amounts of land clearing, there are still relatively large intact areas of native vegetation. Edges may be less important in this region because the interior mallee vegetation provides sufficient shelter and prey. Nevertheless, it is also possible that edge effects occur closer to the agricultural boundary than we sampled (i.e. <2 km).

The use of roads and tracks by foxes is also well documented [51], [52]. Foxes have been found to be more abundant along roadsides [33]. In the mallee system, however, we found similar reporting rates at varying distances (28–1044 m) from roads, indicating foxes use areas well away from roads equally as often as sites close to roads. One hypothesis for the use of roads by foxes is that they provide ‘runways’ which facilitate movement and allow access to foraging areas that would be otherwise difficult to reach [51], [52]. In contrast to environments with a dense understory, mallee vegetation is relatively open, and is unlikely to limit the movement of foxes to roadsides. This may explain the lack of preference for sites near roads in the current study.

### Implications

Fire is used as a conservation tool in Australia and around the world [25]. This study suggests it is unlikely that any particular approach to fire management will alter the reporting rate of the red fox in the semi-arid mallee systems of Australia. However, the presence of foxes in recently burned sites and landscapes is a concern. Predation by invasive mesopredators has been hypothesized as a cause of low post-fire survival in reptiles [14] and mammals [12], [13], due to the reduced cover available in burnt habitats. Although we found no effect of fire history on red fox occurrence, it is possible that predation pressure differs across fire ages due to increased predation risk in recently burned areas. Thus, assessing predation pressure directly across a range of post-fire ages is an important area for further research.

The loss of apex predators can cause smaller predators to increase in abundance, expand their range, and change their temporal activity; this is known as ‘mesopredator release’ [2], [6]. Red foxes have been shown to select particular habitats which may allow them to avoid dominant predators (e.g. coyotes; [53]). As such, one further explanation for the lack of obvious habitat selection by foxes in this system may be the lack of regulating predators. In other Australian systems, the presence of the dingo, Australia’s largest terrestrial apex predator, has been shown to affect fox distributions [3]. Dingoes are largely extinct from the study area but were once common, and as such there is no direct regulation of the abundance or distribution of foxes via biotic interactions. Thus, one potential way to control red foxes in mallee communities is by reinstating dingoes as the apex predator. As this is likely to be a controversial idea owing to the proximity of mallee vegetation to agricultural land and livestock, trialing reintroductions in a controlled and experimental way would be an important first step towards a proof of concept, and a potential solution to this complex conservation issue.

## Supporting Information

Table S1 **Landscape-scale data on the distribution of red foxes in semi-arid land mosaics.**
(DOCX)
