# Supplementary material for: Does Fire Influence the Landscape-Scale Distribution of an Invasive Mesopredator?
Source: PLoS One. 2014 Oct 7;9(10):e107862. doi: 10.1371/journal.pone.0107862 (PMC4188561; doi:10.1371/journal.pone.0107862)
Supplement: Table S1 — Landscape-scale data on the distribution of red foxes in semi-arid land mosaics. (DOCX) [file pone.0107862.s001.docx]

| mosaic | Solar Radiation | Extent of Triodia Mallee | Extent of recently burned | Extent of unburned | Fire diversity | Fox present | Fox absent | Distance to ag land |
| --- | --- | --- | --- | --- | --- | --- | --- | --- |
| 1 | 18.451851 | 95.38 | 0.28 | 99.31 | 0.01932256 | 6 | 12 | 3117.96 |
| 2 | 18.378269 | 95.93 | 0 | 84.04 | 0.439006 | 6 | 12 | 2683.41 |
| 3 | 18.39521 | 99.47 | 20.47 | 64.23 | 0.8963708 | 5 | 13 | 2173.06 |
| 4 | 18.3773 | 84.86 | 59.24 | 36.93 | 0.6693676 | 3 | 15 | 9068.14 |
| 5 | 18.53614 | 10.77 | 0 | 81.93 | 0.4636093 | 8 | 10 | 2611.8 |
| 6 | 18.459261 | 58.99 | 0 | 100 | 0 | 4 | 14 | 18118.45 |
| 7 | 18.4382 | 77.05 | 0 | 61.06 | 0.668479 | 0 | 18 | 16933.05 |
| 8 | 18.500549 | 59.19 | 16.97 | 29.27 | 0.9950548 | 8 | 10 | 1959.52 |
| 9 | 18.42388 | 91.53 | 52.29 | 8.6 | 0.9171692 | 6 | 12 | 7607.98 |
| 10 | 18.37719 | 75.01 | 80.16 | 10.68 | 0.6235984 | 6 | 12 | 18370.08 |
| 11 | 18.344931 | 82.98 | 7.93 | 28.28 | 0.846059 | 5 | 13 | 9828.88 |
| 12 | 18.353161 | 1.21 | 0 | 0 | 0 | 0 | 18 | 3218 |
| 13 | 18.779409 | 74.77 | 56.44 | 43.45 | 0.6846676 | 2 | 16 | 35977.47 |
| 14 | 18.763309 | 60.53 | 64.83 | 34.98 | 0.6477346 | 1 | 17 | 29228.24 |
| 15 | 18.80056 | 31.86 | 3.08 | 96.92 | 0.1375121 | 1 | 17 | 26859.53 |
| 16 | 18.83057 | 18.69 | 2.71 | 97.29 | 0.1245122 | 2 | 16 | 19073.01 |
| 17 | 17.99214 | 0 | 0 | 20.67 | 0.5128806 | 4 | 14 | 3202.41 |
| 18 | 18.051531 | 0 | 0 | 4.51 | 0.1848516 | 2 | 16 | 2893.31 |
| 19 | 19.24642 | 71.09 | 68.37 | 13.85 | 0.8175026 | 3 | 15 | 4257 |
| 20 | 19.263849 | 77.64 | 0.39 | 12.06 | 0.3942374 | 4 | 14 | 4124 |
| 21 | 19.293819 | 67.98 | 0 | 40.14 | 0.6769536 | 1 | 17 | 12227.98 |
| 22 | 19.24353 | 93.31 | 0 | 13.96 | 0.4041859 | 3 | 15 | 18370.77 |
| 23 | 19.125401 | 96.62 | 13.98 | 58.16 | 0.946152 | 3 | 15 | 21691.48 |
| 24 | 19.14496 | 94.04 | 3.47 | 75.6 | 0.6349537 | 1 | 17 | 16973.33 |
| 25 | 18.7295 | 81.38 | 7.68 | 18.15 | 0.739903 | 0 | 18 | 1771.59 |
| 26 | 18.77059 | 49.24 | 6.97 | 47.23 | 0.8987482 | 1 | 17 | 5009.22 |
| 27 | 18.97703 | 97.76 | 58.49 | 1.59 | 0.7457735 | 0 | 18 | 2163.78 |
| 28 | 19.092871 | 99.99 | 16.39 | 0 | 0.4461075 | 8 | 10 | 7143.16 |

Table S1
